# Cardiovascular medication adherence testing in patients living with HIV: A single‐centre observational study

**DOI:** 10.1111/hiv.13715

**Published:** 2024-09-24

**Authors:** Joshua Nazareth, Ayobami Adebayo, Muhammad Fahad, Hanfa Karim, Daniel Pan, Shirley Sze, Christopher A. Martin, Jatinder S. Minhas, Dennis Bernieh, Hanad Osman, Phayre Elverstone, Iain Stephenson, Pankaj Gupta, Manish Pareek

**Affiliations:** ^1^ Department of Infection and HIV Medicine University Hospitals of Leicester NHS Trust Leicester UK; ^2^ Department of Respiratory Sciences University of Leicester Leicester UK; ^3^ Leicester NIHR Biomedical Research Centre Leicester UK; ^4^ Development Centre for Population Health University of Leicester Leicester UK; ^5^ Li Ka Shing Centre for Health Information and Discovery, Oxford Big Data Institute University of Oxford Oxford UK; ^6^ WHO Collaborating Centre for Infectious Disease Epidemiology and Control, School of Public Health, Li Ka Shing Faculty of Medicine The University of Hong Kong Hong Kong China; ^7^ Department of Cardiovascular Sciences University of Leicester Leicester UK; ^8^ Cerebral Haemodynamics in Ageing and Stroke Medicine (CHiASM) Research Group, Department of Cardiovascular Sciences University of Leicester Leicester UK; ^9^ Department of Metabolic Diseases and Chemical Pathology University Hospitals of Leicester NHS Trust Leicester UK; ^10^ Diabetes Research Centre, Department of Population Health Sciences University of Leicester Leicester UK; ^11^ NIHR Applied Research Collaboration Leicester General Hospital Leicester UK

**Keywords:** adherence, cardiovascular disease, comorbidity, HIV, statins

## Abstract

**Introduction:**

People with HIV (PWH) are at an increased risk of developing cardiovascular disease (CVD) compared to HIV‐negative individuals. We sought to evaluate the adherence to medications for CVD in PWH and identify factors associated with non‐adherence to these medications.

**Methods:**

We conducted a cross‐sectional study at the University Hospitals of Leicester NHS Trust between 16 April 2019 and 8 November 2022. We recruited consecutive PWH, who were attending a routine follow‐up outpatient appointment and were prescribed at least one medication for CVD. In addition, we included urinary adherence results of patients with samples collected as part of routine clinical care. We used liquid chromatography–tandem mass spectrometry (LC–MS/MS) to assess if their prescribed medications (antihypertensives, diuretics, beta‐blockers, lipid‐lowering agents, antiplatelets, anticoagulants, antidiabetic medications) were present in the participant's urine sample. Multivariable models were used to identify demographic or clinical features that were associated with non‐adherence.

**Results:**

A total of 162 PWH were included in the analysis. Median age was 55 [interquartile range (IQR): 50–61] years, 63% were male, average time living with HIV was 15 years (IQR: 11–19) and the majority (98%) had an undetectable HIV viral load. In approximately one‐third of patients (59/162), at least one prescribed medication of interest was not detected in urine. Non‐adherence to lipid‐lowering agents was common (35/88, 40%). On multivariable logistic regression, the number of prescribed cardiovascular medications, was associated with medication non‐adherence [medication non‐adherence, per one medication increase: adjusted odds ratio (95% confidence interval) = 1.78 (1.34–2.36); *p* < 0.001].

**Conclusion:**

We found sub‐optimal adherence to medications for CVD in PWH. In order to maximize the clinical benefit of statin therapy in PWH, factors requiring consideration include: improved medication adherence, awareness of polypharmacy, educational interventions and quantitative assessment of sub‐optimal adherence through chemical adherence testing.

## INTRODUCTION

The widespread availability of effective antiretroviral therapy (ART) has significantly diminished the risk of death from HIV‐associated opportunistic infections, resulting in a substantial increase in life expectancy of people with HIV (PWH) [1, 2]. As the population of PWH ages, chronic medical conditions, including cardiovascular disease (CVD), have emerged as predominant causes of morbidity and mortality [2, 3].

The incidence of CVD in PWH is approximately double that of age‐matched HIV‐uninfected individuals [2]. This has been attributed to higher prevalence of cardiovascular risk factors such as smoking, cumulative side‐effects associated with exposure to some antiretroviral agents, and chronic inflammation and persistent immune activation associated with HIV infection [1, 2]. Despite having a higher risk for developing CVD compared with HIV‐negative individuals, PWH often fail to achieve the evidence‐based treatment goals for the prevention of CVD, and the reasons for this are currently poorly understood [4]. In addition, assessing risk in PWH is challenging as calculators for CVD are likely to underestimate the risk in this population [5, 6]. The REPRIVE study recently demonstrated the benefit of pitavastatin in PWH with low‐to‐moderate risk of CVD [7]; therefore, statin use is likely to increase in this patient population.

Medication non‐adherence has become increasingly identified as a major risk factor for treatment failures and poor outcomes, and when this is undiagnosed, it often results in inappropriate escalation of treatment in patients [8]. Non‐adherence is difficult to detect and monitor as the methods used to determine adherence are often limited in terms of reliability and objectivity [9]. Previous studies in PWH have focused on adherence to ART; there is a lack of data on adherence to cardiovascular medications in this population.

We therefore aimed to objectively assess the prevalence of non‐adherence to cardiovascular medications in PWH attending a specialist HIV clinic using liquid chromatography–tandem mass spectrometry (LC–MS/MS) analysis of spot urine samples to detect prescribed cardiovascular medications. We also sought to identify the factors associated with non‐adherence to all prescribed cardiovascular medications, including lipid‐lowering agents.

## METHODS

### Study design

We have used data from two sources in our analysis: the first is a cross‐sectional study and the second is an evaluation of samples collected as part of routine clinical care.

We conducted a cross‐sectional study at the University Hospitals of Leicester (UHL) NHS Trust between 16 April 2019 and 8 November 2022. We recruited consecutive PWH, aged 16 or over, who attended a routine follow‐up outpatient appointment at the Department of Infection and HIV Medicine and were prescribed at least one of the 70 cardiovascular medications which are able to be detected using the LC–MS/MS method [10]. Eligible participants were informed of the study by their clinician at the end of their clinic appointment. Participants were not informed of the study prior to their clinic appointment to prevent potential modification in medication‐taking behaviour. A member of the research team recruited participants after their clinic appointment and participants provided a single urine sample for biochemical analysis.

Urinary adherence analysis was taken up as part of routine clinical care in our patients from 21 February 2023; thus, patients taking part in the study following this date did not require consent, and ethical approval was obtained from a separate protocol for review of our PLHIV research database.

### Demographic and clinical data collection

We collected data on self‐reported age, sex, ethnicity, country of birth, with additional details from patients' clinic letters including number of years living with HIV, their prescribed medications, smoking status, current weight, glycated haemoglobin (HbA1C), low‐density lipoprotein (LDL) cholesterol, HIV viral load and blood pressure measured at the outpatient clinic.

### Ethics

The cross‐sectional study had ethical approval from the Nottingham Research Ethics Committee (REC reference: 17/EM/0027). Participants gave written informed consent prior to any study procedures.

The research database contains routinely collected anonymized patient data of PWH at UHL, for the purpose of evaluating comorbidities in PWH. The database had ethical approval from HRA and Health and Care Research Wales (HCRW) (REC reference: 24/HRA/0348). Both studies were conducted in accordance with ICH‐GCP, Declaration of Helsinki and Data Protection Act 1998 and NHS Act 2006.

### Urine analysis

To assess if the prescribed medication or its metabolite was present in the participant's urine sample, we used LC–MS/MS as previously described [9]. Using this method, we are able to detect the presence of angiotensin‐converting enzyme inhibitors (ACEi), angiotensin receptor blocker (ARB), calcium channel blockers (CCBs), diuretics, alpha‐blockers, beta‐blockers, lipid‐lowering agents, anticoagulants, antidiabetics and antiplatelet medications in urine. Adherence to a prescribed medication was defined as the detection of that medication, or its attributable metabolite, above the assay's lower limit of detection.

### Statistical analysis

We summarized continuous variables that were non‐normally distributed as median with interquartile range (IQR), normally distributed continuous variables as mean and standard deviation (SD) and categorical variables as frequency and percentage. Continuous variables were assessed for normality of distribution by visual inspection. We calculated adherence rates to several classes of cardiovascular medications (Table 1) and summarized these as frequency and percentage.

**TABLE 1:** Complete list of cardiovascular medications by drug class prescribed in this patient cohort that were able to be detected using the liquid chromatography–tandem mass spectrometry method.

ACEi	ARB	CCB	Diuretic	Beta‐blocker	Alpha‐blocker	Lipid	Anticoag	Anti‐DM	Anti‐plt
Lisinopril	Candesartan	Amlodipine	Bendroflumethiazide	Bisoprolol	Doxazosin	Atorvastatin	Warfarin	Empagliflozin	Clopidogrel
Ramipril	Irbesartan	Lacidipine	Spironolactone	Propranolol	Terazosin	Rosuvastatin	Rivaroxaban	Linagliptin	
	Losartan	Nifedipine	Indapamide			Ezetimibe		Metformin	
			Furosemide					Gliclazide	
			Hydrochlorothiazide						

Abbreviations: ACEi, angiotensin‐converting enzyme inhibitor; anticoag, oral anticoagulant; anti‐DM, anti‐diabetes melitus medication; anti‐plt, antiplatelet medication; ARB, angiotensin receptor blocker; CCB, calcium channel blocker.

As in our previous work on medication adherence, we defined medication non‐adherence as complete absence of at least one of the prescribed cardiovascular medications (or their metabolites, where appropriate) in the urine sample analysis [11]. We aimed to explore if either the number of cardiovascular medications or the number of other medications, that were not screened for in the urine such as antidepressants and antiretrovirals, were associated with medication non‐adherence. We therefore used two separate continuous independent variables for total number of prescribed cardiovascular medications (from Table 1) and other medications. We used χ^2^, Wilcoxon rank‐sum and Student's *t*‐tests to compare differences between adherent and non‐adherent by demographic and clinical features. We performed multivariable logistic regression analysis to examine associations with non‐adherence following adjustment for age, sex, ethnicity, number of cardiovascular and other medications. We chose confounders a priori based on previous adherence work in other patient groups [12]. The outcome variable was non‐adherence defined as absence of at least one prescribed medication or the drug metabolite in the urine. We completed subgroup analysis for samples collected as part of the cross‐sectional study and routine clinical care to examine if results varied by study setting.

For the analysis of adherence to lipid‐lowering agents, we performed multivariable logistic regression models adjusting for age, sex, ethnicity, number of cardiovascular and other medications with an outcome variable of absence of at least one prescribed lipid‐lowering agent (ezetimibe and statins) in the urine. We used Student's *t*‐tests to compare differences in LDL cholesterol between those that were adherent and those that were non‐adherent to lipid‐lowering agents.

All analyses were performed using Stata 17 (StataCorp, College Station, Texas, USA). Figures were created in GraphPad Prism version 9.4.1 for macOS (GraphPad Software, San Diego, California USA, www.graphpad.com). We considered *p*‐values <0.05 to be statistically significant.

## RESULTS

A total of 162 PWH were included in the analysis, 99 from the cross‐sectional study and 63 from routine clinical care. Figure S1 shows the formation of the analysed cohort and Table 2 summarizes the demographic and clinical features of the analysed cohort stratified by study setting. We found 98% (158/162) of patients had an undetectable viral load. Overall median age was 55 (IQR: 50–61) years, 63% were male and average years living with HIV was 15 years (IQR: 11–19), which is reflective of the demographics of PWH seen at the trust.

**TABLE 2:** Demographic and clinical features of the analysed cohort stratified by study setting.

	Cross‐sectional study (*n* = 99)	Routine clinical care (*n* = 63)	Total (*n* = 162)	Missing
Median age (IQR)	54 (49–60)	56 (50–63)	55 (50–61)	0
Sex (%)				0
Male	63 (63)	34 (54)	97 (60)	
Female	36 (36)	29 (46)	65 (40)	
Ethnicity (%)				1
White	33 (33)	22 (35)	55 (34)	
Black	53 (54)	27 (43)	80 (50)	
South Asian	10 (10)	9 (15)	19 (12)	
Other/mixed	3 (3)	4 (6)	7 (4)	
Country of birth (%)				26
UK	29 (37)	22 (38)	51 (38)	
Non‐UK	49 (63)	36 (62)	85 (62)	
Time living with HIV (IQR) (years)	15 (11–19)	17 (12–20)	16 (11–19)	7
Weight (IQR) (kg)	88 (74–104)	90 (78–101)	88 (75–102)	4
Smoking status (%)				6
Current	17 (17)	7 (12)	24 (15)	
Ex‐smoker	26 (26)	8 (14)	34 (22)	
Never	56 (56)	42 (74)	98 (63)	
Systolic BP (IQR)	142 (130–154)	138 (130–157)	140 (130–154)	7
HbA1C (IQR)	5.8 (5.4–6.2)	5.8 (5.5–6.3)	5.8 (5.5–6.3)	5
LDL cholesterol (IQR)	2.4 (2.0–3.1)	2.2 (1.7–2.7)	2.3 (1.8–2.9)	7
Number of cardiovascular medications (IQR)	2 (1–3)	2 (2–4)	2 (1–3)	0
Number of other medications (SD)	2 (1–3)	1 (3–4)	2 (1–4)	0
Detectable HIV viral load (%)	2 (2)	2 (3)	4 (2)	0

Abbreviations: BP, blood pressure; HbA1C, haemoglobin A1C; IQR, interquartile range; LDL, low‐density lipoprotein; SD, standard deviation.

**FIGURE 1:**
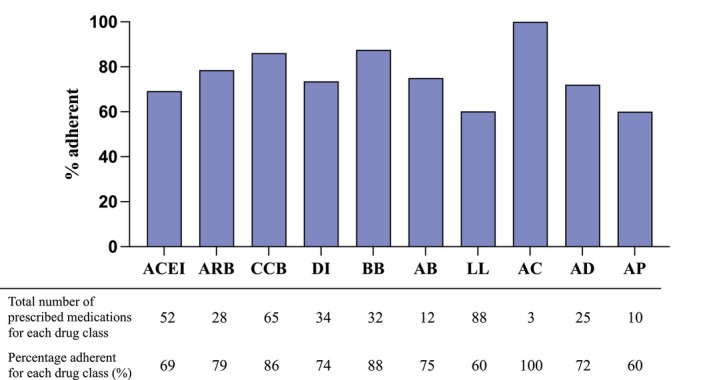
Adherence rates to cardiovascular medications using the liquid chromatography–tandem mass spectrometry method. ACEI, ACE inhibitor; ARB, angiotensin receptor blocker; CCB, calcium channel blocker; Di, diuretic; BB, beta‐blocker; AB, alpha‐blocker; LL, lipid‐lowering agent; AC, anticoagulant; AD, antidiabetic. AP, antiplatelet.

Overall, of the total of 162 PWH, 59 (36%) were non‐adherent to at least one of their prescribed cardiovascular medications. The remaining 103 (64%) had all prescribed cardiovascular medications of interest detectable in urine. Figure 1 summarizes the adherence rates to cardiovascular medications. The highest rates of non‐adherence in this cohort were for antiplatelet (4/10, 40%) and lipid‐lowering agents (35/88, 40%).

Demographic and clinical features of the analysed cohort, stratified by medication non‐adherence, are summarized in Table 3. Individuals that were non‐adherent were prescribed more cardiovascular medications [number of prescribed medications (IQR): adherent, 2 (1–3); non‐adherent, 3 (2–4); *p* < 0.001]. For all other factors we found no differences between the adherent and non‐adherent groups. Similar results were also seen for differences between adherence for both the cross‐sectional study and samples collected as part of routine clinical care (Table S1).

**TABLE 3:** Demographic and clinical features of the analysed cohort stratified by medication non‐adherence.

	Adherent (*n* = 103)	Not‐fully adherent (*n* = 59)	*p*‐value	Total (*n* = 162)
Median age (IQR)	55 (50–61)	55 (49–61)	0.65	55 (50–61)
Sex (%)				
Male	61 (59)	36 (61)	0.82	97 (60)
Female	42 (41)	23 (39)		65 (40)
Ethnicity (%)				
White	37 (34)	18 (31)	0.68	55 (34)
Black	51 (50)	29 (50)		80 (50)
South Asian	10 (10)	9 (16)		19 (12)
Other/mixed	5 (5)	2 (3)		7 (4)
Country of birth (%)				
UK	35 (41)	16 (31)	0.25	51 (38)
Non‐UK	50 (59)	35 (69)		85 (63)
Time living with HIV (IQR) (years)	16 (12–19)	15 (11–19)	0.77	15 (11–19)
Weight (IQR) (kg)	90 (75–102)	88 (75–102)	0.69	88 (75–102)
Smoking status (%)				
Current	14 (14)	10 (18)	0.37	24 (15)
Ex‐smoker	25 (25)	9 (16)		34 (22)
Never	60 (60)	38 (67)		98 (63)
Systolic BP (IQR)	139 (130–151)	143 (132–158)	0.29	140 (130–154)
HbA1C (IQR)	5.7 (5.5–6.2)	6.0 (5.5–6.3)	0.20	5.8 (5.5–6.3)
LDL cholesterol (IQR)	2.3 (1.9–3.0)	2.4 (1.7–2.9)	0.86	2.3 (1.8–2.9)
Number of cardiovascular medications (IQR)	2 (1–3)	3 (2–4)	<0.001	2 (1–3)
Number of other medications (SD)	2 (1–4)	2 (2–4)	0.52	2 (1–4)
Detectable HIV viral load (%)	2 (2)	2 (3)		4 (2)

Abbreviations: BP, blood pressure; HbA1C, haemoglobin A1C; IQR, interquartile range; LDL, low‐density lipoprotein; SD, standard deviation.

**FIGURE 2:**
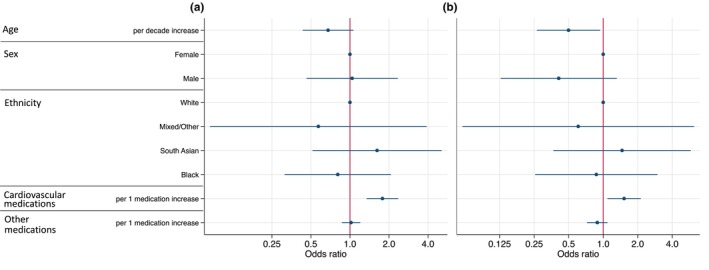
(a, b) Multivariable logistic regression models adjusting for all variables shown with the outcome of medication non‐adherence that is at least one prescribed medication not detected in the urine (a) and non‐adherence to a lipid‐lowering agent (b). Lines represent the 95% confidence intervals for each adjusted odds ratio.

On multivariable logistic regression, the number of cardiovascular medications prescribed was independently associated with overall medication non‐adherence [per one medication increase: adjusted odds ratio (aOR) (95% confidence interval, CI) = 1.78 (1.34–2.36); *p <* 0.001] (Figure 2a). Similar results were also seen for multivariable models completed for each study type (Figure S2). In the multivariable model for non‐adherence to lipid‐lowering agents, the number of cardiovascular medications prescribed was independently associated with non‐adherence to lipid‐lowering agents [per one medication increase (aOR) (95% CI) = 1.52 (1.09–2.12), *p <* 0.01] (Figure 2b). For both non‐adherence to lipid‐lowering agents and overall non‐adherence, there was a trend towards increasing age being inversely associated with non‐adherence [non‐adherence to lipid‐lowering agents, per decade increase: aOR (95% CI) = 0.50 (0.26–0.94), *p =* 0.03; medication non‐adherence, per decade increase: aOR (95% CI) = 0.68 (0.43–1.06), *p =* 0.09] (Figure 2). Individuals who were non‐adherent to lipid‐lowering agents had significantly higher LDL cholesterol levels compared with individuals that were adherent to their lipid‐lowering medications (LDL cholesterol mmol/L (95% CI): adherent, 2.0 (1.8–2.2); non‐adherent, 2.4 (2.0–2.7); *p =* 0.04).

## DISCUSSION

This is the first study, to our knowledge, to examine non‐adherence to cardiovascular medications in PWH using a direct objective measure of non‐adherence, through chemical adherence testing (CAT). Our study found that one in three PWH are not fully compliant with their cardiovascular medications despite demonstrating a high level of adherence to their ART. Our results also demonstrated that a higher number of prescribed medications and decreasing age were associated with medication non‐adherence.

There is increasing evidence in the literature to support the use of the LC–MS/MS technology as a cost‐effective method for diagnosing non‐adherence to cardiovascular medications [13, 14, 15], and this is the first study, to our knowledge, to use this method in PWH. Compared with other methods of screening for adherence, such as patient self‐reporting, pill counting, direct observation, electronic monitoring devices and pharmacy refill data, this method is more accurate, practical and non‐invasive [16]. The stability of cardiovascular medications and its metabolites for this technology has been studied and confirmed. The method has been reported to have high sensitivity and specificity, with medications detectable for four to six half‐lives [11, 17, 18, 19].

Although there were initial concerns expressed about the potential impact of CAT on trust between patients and health providers, a study investigating the attitudes of patients and their clinicians to CAT found that it was well accepted as an effective tool for use in clinical practice by both groups, and it was generally agreed that it could help to overcome issues surrounding adherence if the problems identified through the results are addressed in a polite, non‐judgmental, patient‐centred manner [20].

The present study highlights the sub‐optimal adherence to cardiovascular medications in PWH. Previous adherence studies using alternative approaches such as questionnaire or medication usage data from repeat prescriptions in PWH found similar results with poor adherence to antidiabetics [21], statins [22], and antihypertensive medications [23, 24]. The reasons for this are not well understood; low perceived risk of CVD and challenges in primary care coordination are two reasons that have been suggested in a recent qualitative study [4]. We have also shown that LDL cholesterol is lower in individuals who are non‐adherent to their prescribed lipid‐lowering agents than in those who are adherent. Due to a lack of data on baseline LDL cholesterol and the indication for a lipid‐lowering agent that is for primary or secondary prevention, we are unable to determine whether adherence profiles differed between those who achieved their LDL cholesterol goal and those who did not. Our future work will explore the use of CAT in PWH who are not achieving their cardiovascular disease treatment goals, to gain a better understanding of the role non‐adherence plays in this patient group.

The poor adherence to lipid‐lowering agents we observed is particularly concerning, as the use of these medications is likely to become increasingly common. Following the results of the REPRIVE study, the British HIV Association has recently updated its guidance on prescribing statins; PWH over 40 years old should now all be offered a statin irrespective of lipid profile or estimated CVD risk [7, 25]. The guidance also highlighted the importance of reviewing and addressing medication adherence, especially given the additional pill burden [25]. The reason for the low adherence to lipid‐lowering agents compared with other CVD medications in this cohort is unclear and has also been a finding in other adherence studies [26, 27]. This may reflect a lack of knowledge regarding the indication for this medication and the poor perceptions of the side‐effect profile of statins, and the fact that dyslipidaemia is an asymptomatic condition [28].

At our centre, CAT has prevented unnecessary increases in dosage, or the addition of second‐line agents being prescribed, when patients were found to be non‐adherent. We plan to continue to utilize CAT to address the sub‐optimal adherence to cardiovascular medications seen in this cohort, especially in those who do not achieve treatment targets. Integration of CAT into regular clinic visits will allow for more tailored discussions with patients about their medication‐taking behaviours, provide an opportunity for education regarding the importance of these medications and implement personalized treatment regimens that better suit patients' needs. In future work, we aim to monitor the impact that CAT has on improving the control of cardiometabolic comorbidities and identify the specific barriers to adherence in this patient group.

Notably, our study identified two factors associated with medication non‐adherence in PWH: the number of prescribed cardiovascular medications and decreasing age. These are important findings as they offer the potential to inform targeted strategies to address non‐adherence. Focused strategies have been shown to be the most effective means of improving medication adherence [29]. From our previous work in other patient groups, the number of prescribed medications was also strongly associated with non‐adherence [9, 12]. Given that this was a cross‐sectional study, we are not able to make conclusions regarding the causality. Although it is likely that a high pill burden increases the risk of non‐adherence, there is also the possibility that non‐adherence results in the prescription of more medications to reach treatment goals. Independent of causality, this association is an important finding as strategies such as increased use of fixed‐dose combinations could be utilized to improve adherence, and have been shown to be effective in patients with hypertension [30]. Previous evidence examining the association between adherence and age is variable; some studies have shown that non‐adherence is more common in young people [31, 32], while others have reported the reverse [33] and some studies have shown a non‐linear relationship [34]. Numerous studies have found that females have higher rates of non‐adherence compared with males [35, 36, 37]. A recent study has also shown sub‐optimal achievement of cardiometabolic disease targets in women with HIV [38]. In our cohort we found no significant association between sex and non‐adherence. Further work is required to identify PWH at high risk of non‐adherence and to evaluate the association that this has with achievement of disease targets.

The present study has some limitations. First, patients who could not, or declined to, provide urine samples were excluded from this study, and this may have introduced some bias as these patients may have a different adherence profile from those who participated in the study. Second, utilizing a single spot urine analysis to screen for adherence may not provide a full picture of long‐term adherence in patients and might underestimate chronic non‐adherence as well as intermittent adherence. This is particularly relevant for loop diuretics, which patients often withhold prior to clinic appointments due to increased urinary frequency. Only four patients were prescribed furosemide in this study, with one patient not having the metabolite detected in the urine; inclusion of patients on furosemide would therefore be unlikely to have impacted our overall findings. Other aspects of adherence that were also not able to be assessed in this study included whether the patient adhered to the recommended timing of administration and correct dosage. Lastly, there are several other potential factors associated with adherence that we did not evaluate, such as lifestyle factors, illness beliefs and indication for prescription (e.g. primary or secondary CVD prevention). Further research is needed to explore these associations in order to inform strategies that optimize adherence.

In conclusion, this study offers a unique perspective into the burden of non‐adherence to cardiovascular medications in PWH using the objective method of directly detecting prescribed cardiovascular medications in urine through the use of the LC–MS/MS technology. We identified a high prevalence of non‐adherence to cardiovascular medications among PWH, which is troubling given the widely recognized increased risk of cardiovascular diseases in this patient group. To maximize the effectiveness of cardiovascular medications in PWH, careful consideration should be made to improve medication adherence. This could include the use of polypills to decrease pill burden, educational interventions and identifying sub‐optimal adherence, such as utilizing CAT.

## AUTHOR CONTRIBUTIONS

JN contributed to data collection, data analysis, manuscript drafting and editing. AA contributed to data collection, data analysis and manuscript drafting. FM, HK, PE and IS contributed to data collection and manuscript editing. DP, SS, CAM, JSM and MP contributed to data analysis and manuscript editing. DB, HO and PG contributed to study design, data collection, manuscript drafting and editing.

## FUNDING INFORMATION

The study required no additional funding. MP is supported by the NIHR Leicester Biomedical Research Centre (BRC) and National Institute for Health and Care Research (NIHR) Applied Research Collaboration East Midlands (ARC EM). DP is supported by a NIHR Doctoral Research Fellowship (NIHR302338). HO and PG are supported by the NIHR Leicester BRC.

## CONFLICT OF INTEREST STATEMENT

MP reports grants from UKRI‐MRC for the current work, and UKRI‐MRC, NIHR, Sanofi, Gilead and Moderna outside the current work, and has received consulting fees from QIAGEN. DP is supported by a NIHR Doctoral Research Fellowship. SS is supported by NIHR clinical lectureship. JSM is supported by a Stroke Association Senior Clinical Lectureship (SA SCLM23\100 003) and UKRI Future Leaders Fellowship (MR/Y016807/1). The views expressed are those of the authors and not necessarily those of the NIHR, UKRI, Stroke Association or the Department of Health and Social Care. HO is funded by Servier Affaires Medical. Servier Affaires Medical was not involved in any aspect of the study. IS reports educational support from ViiV, MSD and Gilead for conference registration and has received fees for advisory board participation from ViiV and Gilead. The other authors report no conflicts of interest.

## ETHICS STATEMENT

The cross‐sectional study had ethical approval from the Nottingham Research Ethics Committee (REC reference: 17/EM/0027). Participants gave written informed consent prior to any study procedures. The research database contains routinely collected anonymized patient data of PWH at UHL, for the purpose of evaluating comorbidities in PWH. The database had ethical approval from HRA and Health and Care Research Wales (HCRW) (REC reference: 24/HRA/0348). Both studies were conducted in accordance with ICH‐GCP, Declaration of Helsinki and Data Protection Act 1998 and NHS Act 2006.

## Supporting information


**Figure S1.** Flowchart depicting the number of patients from the cross‐sectional observational study and routine clinical care included in the final analysis.

**Figure S2.** Multivariable logistic regression models adjusting for all variables shown with the outcome of medication non‐adherence that is at least one prescribed medication not detected in the urine for results from the cross‐sectional study (a) and routine clinical care (b). Lines represent the 95% confidence intervals for each adjusted odds ratio.

**Table S1.** Demographic and clinical features of the analysed cohort stratified by medication non‐adherence for each study type. IQR, interquartile range; s.d, standard deviation; BP, blood pressure; HbA1C, haemoglobin A1C; LDL low‐density lipoprotein.

## References

[1] Maggi P , De Socio GV , Cicalini S , et al. Statins and aspirin in the prevention of cardiovascular disease among HIV‐positive patients between controversies and unmet needs: review of the literature and suggestions for a friendly use. AIDS Res Ther. 2019;16(1):11. 31126301 10.1186/s12981-019-0226-2 PMC6534832

[2] Martin‐Iguacel R , Llibre JM , Friis‐Moller N . Risk of cardiovascular disease in an aging HIV population: where are we now? Curr HIV/AIDS Rep. 2015;12(4):375‐387. 26423407 10.1007/s11904-015-0284-6

[3] Shah A , Martin V , Brown A , et al. Mortality among people with HIV in the UK in 2021: findings from the national HIV mortality review. HIV Med. 2023;24(S3):21‐106.

[4] Muiruri C , Sico IP , Schexnayder J , et al. Why do people living with HIV adhere to antiretroviral therapy and not comorbid cardiovascular disease medications? A Qualitative Inquiry. Patient Prefer Adherence. 2020;14:985‐994. 32669837 10.2147/PPA.S254882 PMC7337208

[5] Achhra AC , Lyass A , Borowsky L , et al. Assessing cardiovascular risk in people living with HIV: current tools and limitations. Curr HIV/AIDS Rep. 2021;18(4):271‐279. 34247329 10.1007/s11904-021-00567-w PMC8733948

[6] Triant VA , Perez J , Regan S , et al. Cardiovascular risk prediction functions underestimate risk in HIV infection. Circulation. 2018;137(21):2203‐2214. 29444987 10.1161/CIRCULATIONAHA.117.028975 PMC6157923

[7] Grinspoon SK , Fitch KV , Zanni MV , et al. Pitavastatin to prevent cardiovascular disease in HIV infection. N Engl J Med. 2023;389:687‐699. 37486775 10.1056/NEJMoa2304146 PMC10564556

[8] Kolandaivelu K , Leiden BB , O'Gara PT , Bhatt DL . Non‐adherence to cardiovascular medications. Eur Heart J. 2014;35(46):3267‐3276. 25265973 10.1093/eurheartj/ehu364

[9] Lane D , Beishon L , Sharma V , et al. High non‐adherence rates to secondary prevention by chemical adherence testing in patients with TIA. J Stroke Cerebrovasc Dis. 2022;31(9):106665. 35901588 10.1016/j.jstrokecerebrovasdis.2022.106665

[10] Osman H , Lane D , Bernieh D , et al. An innovative chemical adherence test demonstrates very high rates of nonadherence to Oral cardio‐metabolic medications. Kidney Int Rep. 2023;8(12):2818‐2821. 38106590 10.1016/j.ekir.2023.09.033 PMC10719591

[11] Tomaszewski M , White C , Patel P , et al. High rates of non‐adherence to antihypertensive treatment revealed by high‐performance liquid chromatography‐tandem mass spectrometry (HP LC‐MS/MS) urine analysis. Heart. 2014;100(11):855‐861. 24694797 10.1136/heartjnl-2013-305063 PMC4033175

[12] Gupta P , Patel P , Štrauch B , et al. Risk factors for nonadherence to antihypertensive treatment. Hypertension. 2017;69(6):1113‐1120. 28461599 10.1161/HYPERTENSIONAHA.116.08729

[13] Lane D , Patel P , Khunti K , Gupta P . Objective measures of non‐adherence in cardiometabolic diseases: a review focused on urine biochemical screening. Patient Prefer Adherence. 2019;13:537‐547. 31043772 10.2147/PPA.S162215 PMC6469740

[14] Osman H , Alghamdi R , Gupta P . Review of the methods to measure non‐adherence with a focus on chemical adherence testing. Translational Metabolic Syndrome Research. 2021;5:5‐9.

[15] van Schoonhoven AV , van Asselt ADI , Tomaszewski M , et al. Cost‐utility of an objective biochemical measure to improve adherence to antihypertensive treatment. Hypertension. 2018;72(5):1117‐1124. 30354817 10.1161/HYPERTENSIONAHA.118.11227

[16] Gupta P , Patel P , Horne R , Buchanan H , Williams B , Tomaszewski M . How to screen for non‐adherence to antihypertensive therapy. Curr Hypertens Rep. 2016;18(12):89. 27889904 10.1007/s11906-016-0697-7 PMC5124437

[17] Murray GJ , Danaceau JP . Simultaneous extraction and screening of diuretics, beta‐blockers, selected stimulants and steroids in human urine by HPLC‐MS/MS and UPLC‐MS/MS. J Chromatogr B Analyt Technol Biomed Life Sci. 2009;877(30):3857‐3864. 10.1016/j.jchromb.2009.09.036 19837636

[18] Lawson AJ , Shipman KE , George S , Dasgupta I . A novel ‘Dilute‐and‐Shoot’ liquid chromatography‐tandem mass spectrometry method for the screening of antihypertensive drugs in urine. J Anal Toxicol. 2016;40(1):17‐27. 26333988 10.1093/jat/bkv102

[19] Burns AD , Lane D , Cole R , Patel P , Gupta P . Cardiovascular medication stability in urine for non‐adherence screening by LC‐MS‐MS. J Anal Toxicol. 2019;43(4):325‐329. 30517637 10.1093/jat/bky090

[20] Schesing KB , Chia R , Elwood B , et al. Assessment of patient and provider attitudes towards therapeutic drug monitoring to improve medication adherence in low‐income patients with hypertension: a qualitative study. BMJ Open. 2020;10(11):e039940. 10.1136/bmjopen-2020-039940 PMC7703422 33247015

[21] Batchelder AW , Gonzalez JS , Berg KM . Differential medication nonadherence and illness beliefs in co‐morbid HIV and type 2 diabetes. J Behav Med. 2014;37(2):266‐275. 23277233 10.1007/s10865-012-9486-1

[22] Crockett KB , Wen Y , Overton ET , et al. One‐year statin persistence and adherence in adults with HIV in the United States. J Clin Lipidol. 2021;15(1):181‐191. 33341376 10.1016/j.jacl.2020.11.001 PMC7887025

[23] Jackson IL , Okonta JM , Ukwe CV . HIV‐ and hypertension‐related knowledge and medication adherence in HIV seropositive persons with hypertension. J Public Health (Oxf). 2022;44(1):e79‐e87. 33348362 10.1093/pubmed/fdaa221

[24] Langness J , Cook PF , Gill J , Boggs R , Netsanet N . Comparison of adherence rates for antiretroviral, blood pressure, or mental health medications for HIV‐positive patients at an academic medical center outpatient pharmacy. J Manag Care Spec Pharm. 2014;20(8):809‐814. 25062074 10.18553/jmcp.2014.20.8.809 PMC10437659

[25] Waters L , Gilleece Y , Alagaratnam J , et al. BHIVA Rapid Guidance on the Use of Statins for Primary Prevention of Cardiovascular Disease in People Living with HIV. British HIV Association; 2024.

[26] Denicolò S , Reinstadler V , Keller F , et al. Non‐adherence to cardiometabolic medication as assessed by LC‐MS/MS in urine and its association with kidney and cardiovascular outcomes in type 2 diabetes mellitus. Diabetologia. 2024;67(7):1283‐1294. 38647650 10.1007/s00125-024-06149-w PMC11153278

[27] Patel P , Gupta P , Burns A , et al. Biochemical urine testing of adherence to cardiovascular medications reveals high rates of nonadherence in people attending their annual review for type 2 diabetes. Diabetes Care. 2019;42(6):1132‐1135. 30885952 10.2337/dc18-1453

[28] Matthews A , Herrett E , Gasparrini A , et al. Impact of statin related media coverage on use of statins: interrupted time series analysis with UK primary care data. BMJ. 2016;353:i3283. 27353418 10.1136/bmj.i3283 PMC4925917

[29] Nieuwlaat R , Wilczynski N , Navarro T , et al. Interventions for enhancing medication adherence. Cochrane Database Syst Rev. 2014;11:CD000011. doi:10.1002/14651858.CD000011.pub4 PMC7263418 25412402

[30] Gupta AK , Arshad S , Poulter NR . Compliance, safety, and effectiveness of fixed‐dose combinations of antihypertensive agents. Hypertension. 2010;55(2):399‐407. 20026768 10.1161/HYPERTENSIONAHA.109.139816

[31] Ge L , Heng BH , Yap CW . Understanding reasons and determinants of medication non‐adherence in community‐dwelling adults: a cross‐sectional study comparing young and older age groups. BMC Health Serv Res. 2023;23(1):905. 37620970 10.1186/s12913-023-09904-8 PMC10464472

[32] Fernandez‐Lazaro CI , García‐González JM , Adams DP , et al. Adherence to treatment and related factors among patients with chronic conditions in primary care: a cross‐sectional study. BMC Fam Pract. 2019;20(1):132. 31521114 10.1186/s12875-019-1019-3 PMC6744672

[33] Degli Esposti L , Saragoni S , Benemei S , et al. Adherence to antihypertensive medications and health outcomes among newly treated hypertensive patients. Clinicoecon Outcomes Res. 2011;3:47‐54. 21935332 10.2147/CEOR.S15619 PMC3169972

[34] Kim SJ , Kwon OD , Han EB , et al. Impact of number of medications and age on adherence to antihypertensive medications: a nationwide population‐based study. Medicine (Baltimore). 2019;98(49):e17825. 31804305 10.1097/MD.0000000000017825 PMC6919523

[35] Vervloet M , Korevaar JC , Leemrijse CJ , Paget J , Zullig LL , van Dijk L . Interventions to improve adherence to cardiovascular medication: what about gender differences? A systematic literature review. Patient Prefer Adherence. 2020;14:2055‐2070. 33154630 10.2147/PPA.S260562 PMC7606362

[36] Lewey J , Shrank WH , Bowry AD , Kilabuk E , Brennan TA , Choudhry NK . Gender and racial disparities in adherence to statin therapy: a meta‐analysis. Am Heart J. 2013;165(5):665‐678.e1. 23622903 10.1016/j.ahj.2013.02.011

[37] Venditti V , Bleve E , Morano S , Filardi T . Gender‐related factors in medication adherence for metabolic and cardiovascular health. Metabolites. 2023;13(10):1087. doi:10.3390/metabo13101087 PMC10609002 37887412

[38] Mazzitelli M , Scaglione V , Cozzolino C , et al. Achievement of primary prevention cardiometabolic targets in women with HIV: an urgent call to action to pursue cardiovascular health. Viruses. 2024;16(4):578. 38675920 10.3390/v16040578 PMC11054919

