# Supplementary material for: Cardiovascular medication adherence testing in patients living with HIV: A single‐centre observational study
Source: HIV Med. 2024 Sep 24;25(12):1330–9. doi: 10.1111/hiv.13715 (PMC11608581; doi:10.1111/hiv.13715)
Supplement: Supplementary file 1 — Figure S1. Flowchart depicting the number of patients from the cross‐sectional observational study and routine clinical care included in the final analysis. [file HIV-25-1330-s003.pdf]

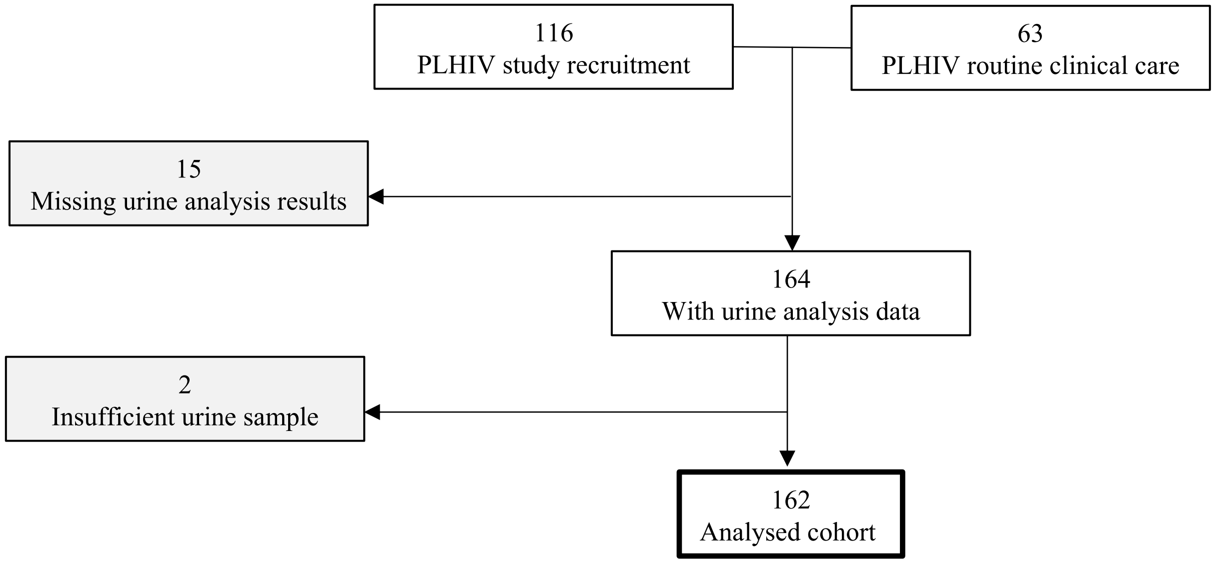

Supplementary Figure 1. Flowchart depicting the number of patients from the cross-sectional observational study and routine clinical care included in the final analysis.
