# Supplementary material for: Cardiovascular medication adherence testing in patients living with HIV: A single‐centre observational study
Source: HIV Med. 2024 Sep 24;25(12):1330–9. doi: 10.1111/hiv.13715 (PMC11608581; doi:10.1111/hiv.13715)
Supplement: Supplementary file 2 — Figure S2. Multivariable logistic regression models adjusting for all variables shown with the outcome of medication non‐adherence that is at least one prescribed medication not detected in the urine for results from the cross‐sectional study (a) and routine clinical care (b). Lines represent the 95% confidence intervals for each adjusted odds ratio. [file HIV-25-1330-s001.pdf]

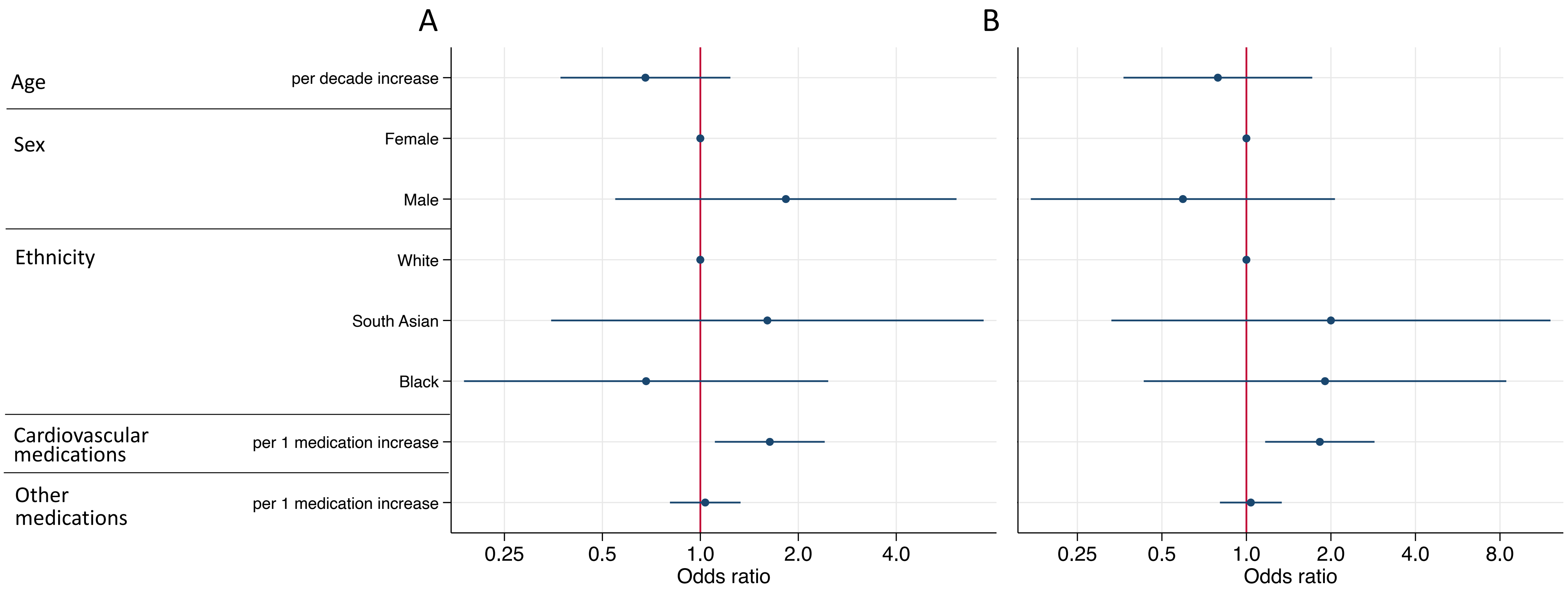

Supplementary Figure 2. Multivariable logistic regression models adjusting for all variables shown with the outcome of medication non-adherence i.e. at least one prescribed medication not detected in the urine for results from (A) the cross-sectional study and (B) routine clinical care. Lines represent the 95% confidence intervals for each adjusted odds ratio.
