# Supplementary material for: Cardiovascular medication adherence testing in patients living with HIV: A single‐centre observational study
Source: HIV Med. 2024 Sep 24;25(12):1330–9. doi: 10.1111/hiv.13715 (PMC11608581; doi:10.1111/hiv.13715)
Supplement: Supplementary file 3 — Table S1. Demographic and clinical features of the analysed cohort stratified by medication non‐adherence for each study type. IQR, interquartile range; s.d, standard deviation; BP, blood pressure; HbA1C, haemoglobin A1C; LDL low‐density lipoprotein. [file HIV-25-1330-s002.pdf]

	Cross-sectional study				Routine clinical care			
	Adherent n=67	Not-fully adherent n=32	*p* value	Total n=99	Adherent n=36	Not-fully adherent n=27	*p* value	Total n=63
Median age (IQR)	54 (49-60)	55 (48-59)	0.72	54 (49-60)	57 (51-64)	55 (50-62)	0.54	56 (50-63)
Sex (%)								
Male	39 (58)	24 (75)	0.10	63 (64)	22 (61)	12 (44)	0.10	34 (54)
Female	28 (42)	8 (25)		36 (36)	14 (39)	15 (56)		29 (46)
Ethnicity (%)								
White	21 (31)	12 (38)	0.32	33 (33)	16 (44)	6 (23)	0.37	22 (35)
Black	38 (57)	15 (47)		53 (54)	13 (36)	14 (54)		27 (44)
South Asian	5 (7)	5 (16)		10 (10)	5 (14)	4 (15)		9 (15)
Other / Mixed	3 (4)	0 (0)		3 (3)	2 (6)	2 (8)		4 (6)
Country of birth (%)								
UK	19 (37)	10 (37)	0.99	29 (37)	16 (47)	6 (25)	0.09	22 (38)
Non-UK	32 (63)	17 (63)		49 (63)	18 (53)	18 (75)		36 (62)
Years living with HIV (IQR)	16 (12-19)	13 (10-18)	0.55	15 (11-19)	16 (10-21)	18 (12-20)	0.93	17 (12-20)
Weight in Kg (IQR)	86 (73-104)	89 (79-98)	0.77	88 (74-104)	91 (79-100)	87 (71-102)	0.34	90 (78-101)
Smoking status (%)								
Current	10 (15)	7 (22)	0.43	17 (17)	4 (13)	3 (12)	0.92	7 (12)
Ex-smoker	20 (30)	6 (19)		26 (26)	5 (16)	3 (12)		8 (14)
Never	37 (55)	19 (59)		56 (57)	23 (72)	19 (76)		42 (74)
Systolic BP (IQR)	140 (128-151)	148 (132-166)	0.03	142 (130-154)	138 (130-157)	138 (128-154)	0.43	138 (130-157)
HbA1C (IQR)	5.7 (5.4-6.1)	5.9 (5.4-6.5)	0.15	5.8 (5.4-6.2)	5.8 (5.5-6.4)	6.0 (5.6-6.3)	0.85	5.8 (5.5-6.3)
LDL Cholesterol (IQR)	2.5 (2.1-3.2)	2.3 (1.7-2.8)	0.44	2.4 (2.0-3.1)	2.1 (1.7-2.6)	2.5 (2.0-2.9)	0.12	2.2 (1.7-2.7)
Number of cardiovascular meds (s.d)	2 (1-3)	2 (1-4)	0.07	2 (1-3)	2 (1-3)	2 (3-4)	0.003	2 (2-4)
Number of other meds (s.d)	2 (1-3)	2 (2-4)	0.57	2 (1-3)	3 (1-4)	2 (2-4)	0.90	3 (1-4)
Detectable HIV Viral load	2 (3)	0	0.32	2 (2)	0	2 (7)	0.10	2 (3)

Supplementary Table 1. Demographic and clinical features of the analysed cohort stratified by medication non-adherence for each study type. IQR, Interquartile range; s.d, Standard deviation; BP, Blood pressure; HbA1C, Haemoglobin A1C; LDL Low density lipoprotein.
